# Chronic Inflammation and Radiation-Induced Cystitis: Molecular Background and Therapeutic Perspectives

**DOI:** 10.3390/cells10010021

**Published:** 2020-12-24

**Authors:** Carole Helissey, Sophie Cavallero, Clément Brossard, Marie Dusaud, Cyrus Chargari, Sabine François

**Affiliations:** 1 Department of Radiation Biological Effects, French Armed Forces Biomedical Research Institute, 91220 Brétigny-sur-Orge, France; carole.helissey@intradef.gouv.fr (C.H.); sophie.cavallero@def.gouv.fr (S.C.); cyrus.chargari@gustaveroussy.fr (C.C.); 2 Clinical Unit Research, HIA Bégin, 94160 Saint-Mandé, France; 3 Radiobiology of Medical Exposure Laboratory (LRMed), Institute for Radiological Protection and Nuclear Safety (IRSN), 92260 Fontenay-aux-Roses, France; clement.brossard@irsn.fr; 4 Department of Urology, HIA Bégin, 94160 Saint-Mand, France; marie.dusaud@gmail.com; 5 Gustave Roussy Comprehensive Cancer Center, Department of Radiation Oncology, 94805 Villejuif, France; 6 French Military Health Academy, Ecole du Val-de-Grâce (EVDG), 75005 Paris, France

**Keywords:** radiation therapy, radiation cystitis, fibrosis, treatment, stem cells therapy, macrophages

## Abstract

Radiation cystitis is a potential complication following the therapeutic irradiation of pelvic cancers. Its clinical management remains unclear, and few preclinical data are available on its underlying pathophysiology. The therapeutic strategy is difficult to establish because few prospective and randomized trials are available. In this review, we report on the clinical presentation and pathophysiology of radiation cystitis. Then we discuss potential therapeutic approaches, with a focus on the immunopathological processes underlying the onset of radiation cystitis, including the fibrotic process. Potential therapeutic avenues for therapeutic modulation will be highlighted, with a focus on the interaction between mesenchymal stromal cells and macrophages for the prevention and treatment of radiation cystitis.

## 1. Introduction

External pelvic radiation therapy is an important tool in the therapeutic arsenal for the treatment of pelvic cancers, such as prostate cancer, cervical cancer, rectal cancer or bladder cancer. Improvements in radiation techniques, such as intensity-modulated radiotherapy (IMRT), stereotactic radiotherapy and image-guided brachytherapy, have made it possible to deliver increasingly effective doses in smaller volumes with a clear improvement in treatment tolerance. However, the bladder is a critical organ thatmay be sensitive to low doses of radiation. Despite improved techniques, pelvic irradiation is still responsible for acute and/or late adverse events affecting the bladder. The term “radiation cystitis” therefore includes all lesions and symptoms of the bladder following the irradiation of the pelvic organs. Its severity is related to the volume of radiation exposure, the total dose delivered as well as the administration schedule and fractionation. This adverse event may have an impact on patients’ quality of life. As cancer patient survival improves, long-term survivorship issues are of increasing importance, and an improved understanding of radiation-induced cystitis mechanisms is essential [1].

In this review, we review the available literature on clinical presentation and pathophysiology of acute and late radiation cystitis. Then, currently available treatments are examined. Due to the lack of long-term clinical benefit, other therapeutic avenues must be developed for the management of this adverse event. Finally, we highlight the place of immunity in the pathological processes of radiation cystitis and its potential as a therapeutic target, focusing on the interaction between Mesenchymal Stromal Cells (MSCs) and macrophages.

## 2. Background Information

The reported incidence of radiation cystitis ranges from 9.1% to 80% [2]. This variability is linked to methods of evaluation and monitoring. Indeed, symptoms of late radiation cystitis may occur very late (sometimes decades) after their therapeutic irradiation, and some patients may be lost to follow-up. Similarly, acute manifestations may be underestimated (and therefore not reported) while irreversible radiation-induced bladder lesions are developing. Late radiation cystitis is the result of an ongoing process of destruction of bladder tissue and histological changes, and a continuum between acute and late radiation lesions do exist.

### 2.1. Acute Radiation Cystitis

**Figure 1:**
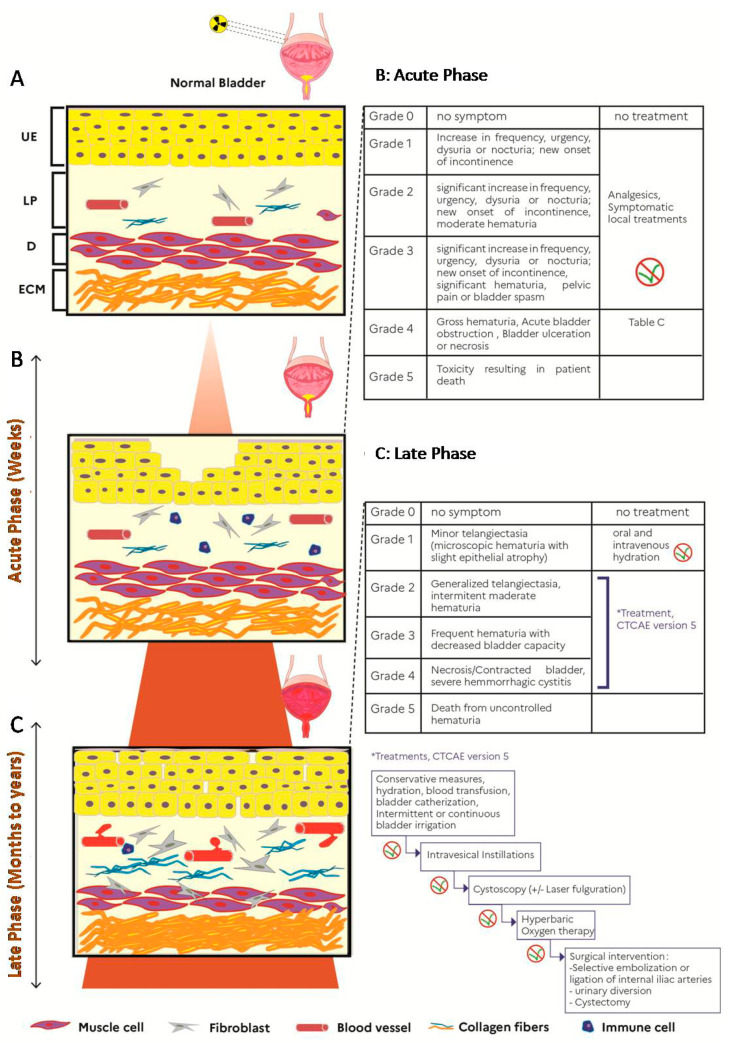
Illustration of radiation cystitis (RC) and clinical management adapted with “Modeling and treatment of radiation cystitis [7], development of RC after radiotherapy (**A**), in the acute phase (infiltration of immune cells into the lamina propria (LP) and depletion of the urothelium (UE)), in latent phase (proliferation of fibroblasts with hematuria, dilation of vessels, bleeding, decrease in the detrusor muscle layer (D) and production of collagen in LP and extracellular matrix(ECM). (F: fibroblast, V: vessel, M: muscle cells, C: collagen fibers, I: immune cells) (**B**) Clinical management during the acute phase of CR. (**C**) Clinical management during the latent phase of CR. * corresponds to cascade treatments of grades 2 to 4 according to CTCAE version 5 [3].

Acute radiation cystitis is defined as any adverse event occurring during or up to threemonths after the end of radiation therapy (the threshold of sixmonths was also proposed). Its incidence is estimated at nearly 50% following pelvic irradiation at full curative doses (e.g., prostate or locally advanced cervical cancer treatment). Clinical symptoms may include increased urinary urgency and frequency (pollakiuria), both during the day and at night, dysuria, but also cystalgia with bladder spasms, and hematuria, albeit rarely at this early stage. An international grade classification ranging from 1 to 5 can be used to assess the severity and impact on the quality of life (Figure 1) [3]. Acute radiation tissue injury to the bladder is caused primarily by damage to the bladder mucosa. It involves an acute inflammatory response and tissue edema. Urothelial regeneration thus comes to a halt, and the epithelium is desquamated with no regeneration, which results in urothelial lesions making the bladder vulnerable to trauma and infections [4]. These lesions are characterized by edema, hyperemia and inflammation of the mucous membrane. In most of the cases, the prognosis is favorable, as these reactions usually disappear spontaneously within fourto sixweeks after the completion of radiation therapy [4,5], but an interruption of radiation therapy may be considered in case of severe grade 3–4 symptoms. Such treatment disruptions may potentially lead to a decrease in tumor control because of an increase in overall treatment time and should, therefore, be discussed on an individual basis [6].

### 2.2. Late Radiation Cystitis

Late radiation cystitis is defined as an adverse event associated with pelvic irradiation that occurs after a minimum of threemonths and possibly even several years after completion of radiation therapy. Toxicities occurring between three and sixmonths are sometimes considered as “early delayed”. On average, late radiation cystitis appears within the following 2–3 years. The incidence of late symptomatic radiation cystitis is stable over time at 5–10%, despite improved radiation techniques [8,9,10]. The clinical presentation can be variable, including bladder pain, urinary urgency, isolated urinary disorders and pollakiuria. Given that these symptoms are nonspecific and appear long after treatment, urine culture, or even cystoscopy may be useful to rule out other differential diagnoses. The most pathognomonic clinical feature is recurrent hematuria, with varying severity. In its most (and rare) severe forms, late radiation cystitis may be life-threatening. The incidence of late radiation cystitis was approximately 5% at 5 years and 10% at 20 years with conventional radiotherapy techniques [9]. It is important to eliminate any local recurrence or new cancer by performing cystoscopy. It should be highlighted that severe late symptoms (e.g., fistulas) may be worsened by inappropriate bladder biopsies, which should therefore be avoided in previously irradiated areas. Patients with a pelvic tumor extending to the bladder are also at high risk of fistulas [11]. A classification of this adverse event was developed (Figure 1). Severe late radiation cystitis is related to the volume and the dose of radiation exposure, the administration schedule and the technique used, but it is also important to identify patients with risk factors for developing a severe form. Marks et al. reported that patients with co-morbidities, such as hypertension, diabetes, a history of abdominal surgery, and patients receiving concomitant chemotherapy were at higher risk of developing radiation cystitis, especially in its late form [12]. Recent data suggested that after high dose exposures (such as after brachytherapy treatment), some anatomic subpart of the bladder may be at higher risk of complication, such as the bladder neck [13]. Although the pathophysiology of late radiation cystitis still remains unclear, endothelial cells appear to play an important role in this mechanism. Indeed, the submucosal vascularity is damaged by fibrosis of the vascular intima resulting in vessel obliteration and submucosal/muscular fibrosis. This is followed by urothelial atrophy, hypoxia with hypovascularization and ischemia of the bladder leading to the development of fibrosis and atrophy of the bladder tissue with the emergence of neovascularization in the form of telangiectasia that may easily bleed [14,15]. At the later stage, reduction in bladder capacity is observed linked to complete bladder fibrosis, mucosal ulcers with the risk of fistulization and spontaneous perforations of the bladder (or fistulae resulting from biopsies).

## 3. Current Treatments and Clinical Trials

### 3.1. Acute and Late Radiation Cystitis with Storage, Voiding Symptoms or Occasional Bleeding

The clinical management of storage symptoms for acute and late radiation cystitis is largely symptomatic with analgesics and anti-inflammatory drugs. Good hydration is recommended for patients in order to increase diuresis, cleanse the bladder, and avoid urinary obstruction resulting from blood clots [16].

Likewise, anticholinergics, like oxybutynin, trospium chloride, solifenacin, fesoterodine or flavoxate hydrochloride, can be prescribed to help alleviate urgency and increased daytime frequency. Their action is to decrease the contractility of the detrusor and improve symptoms [4].

In some cases, antibiotics may be proposed to prevent the condition from worsening in the event of infection.

Alpha-blockers, 5-reductase inhibitors or phosphodiesterase 5 inhibitors may be useful to alleviate voiding symptoms. Their action is to decrease the tone of the posterior urethra, bladder neck and the volume of the prostate [1]. In severe cases, it is sometimes necessary to hospitalize the patient for transfusions or clot evacuation [4]. In fact, bladder irrigations are performed in order to obtain a dilution of hematuria and drain the clots. It is a sterile technique with lubrication for standard catheter insertion with a large three-way catheter. Blood clot evacuation is performed manually by using a large Toomey or catheter syringe until no further clots and output begin to clear. Then, we use normal saline (0.9%) for continuous irrigation [17].

If acute active bleeding does persist and is refractory to irrigations, electrocoagulation should be discussed, as described by Martinez and colleagues [18]. The procedure was performed with a rigid 22 French cystoscope. It was performed to identify the source of bleeding and rule out any other unidentified pathology. The Green Light laser was used to target any active source of bleeding. These areas were coagulated with the laser. Throughout the procedure, saline irrigation was used, and care was taken to ensure that the ureteral orifices were not injured. At the completion of the procedure, the bladder was drained under direct visualization to ensure adequate hemostasis. Very minimal bladder mucosal damage was reported. Then, a large three-way catheter was placed, and continuous irrigation was maintained overnight and stopped the next morning [18].

These treatments are tailored according to the severity of the symptoms (Figure 1).

### 3.2. Late Radiation Cystitis with Persistent or Recurrent Hematuria

#### 3.2.1. Intravesical Instillations

Different molecules have been used for this indication, with different mechanisms of action. Their objectives are sterilization, cleansing and arrest of focal bleeding points.

*Aluminum salt:* Intravesical aluminous salts are considered astringent agents. They exert their action through protein precipitation on the cell surface and in interstitial spaces. They decrease blood vessel diameter and stiffness of capillary endothelium [9,19]. Aluminum salts are typically delivered as a 1% concentration of alum mixed with sterile water. Westerman et al. evaluated the benefit of alum instillations in 40 patients with hematuria, which was linked in 95% of patients to radiation cystitis [20]. These instillations led to a reduction in transfusion requirements (82% before instillation vs. 59% after instillation, *p* = 0.05). Moreover, 32.5% of patients did not require additional treatment after a median follow-up of 17 months. Tolerance was generally good. The main side effect reported was bladder spasm in 35% of patients [20].

*Formalin*: Formalin action consists of precipitating cellular proteins in the mucosa of the bladder. The consequence is to create occlusion within telangiectatic tissue. It appears to be the most effective intravesical agent with complete resolution rates ranging from 70 to 89%. However, the safety profile for this treatment is mediocre. First of all, its instillation is quite painful and must therefore be performed under general anesthesia. In addition, formalin has a high rate of morbidity and mortality (31%), with risks of vesicoureteral reflux complicated by severe bilateral pyelonephritis, ureteral stenosis and fibrosis of the bladder with reduced capacity and increased urinary frequency [21]. To date, its use remains very limited due to its poor safety profile.

*Hyaluronic acid*: Hyaluronic acid is a mucopolysaccharidethathelps to repair the normal glycosaminoglycan layer of the bladder when administrated through intravesical instillations. It has immunomodulatory properties that enhance connective tissue healing. Shao et al. evaluated the efficacy of intravesical hyaluronic acid (HA) instillation and hyperbaric oxygen (HBO) in the management of hemorrhagic radiation cystitis [22]. The clinical benefit was identical in the 2 groups but was maintained over time significantly in the HA arm. Indeed, complete resolution of hematuria was noted in 88%, 75%, and 50% of HA patients and in 75%, 50%, and 45% of patients in the HBO group, at 6-, 12- and 18-months following therapy, respectively. Hyaluronic acid appears to be an interesting therapeutic alternative, though this must be confirmed in a larger cohort.

Other agents have shown interesting results but have been studied only in small cohorts, like botulinum toxin, chondroitin sulfate, polydeoxyribonucleotides, early placental extract [23,24,25,26].

#### 3.2.2. Hyperbaric Oxygen Therapy (HBOT)

This technique consists of placing the patient in a pressurized chamber (hyperbaric chamber) to administer pure or mixed oxygen at a pressure greater than atmospheric pressure, for 5–7 days a week, for a daily duration of 60–90 min up to approximately 30–45 sessions [27]. The effect of hyperbaric oxygen therapy is to allow better oxygen diffusion in tissues and to disrupt the continuum between hypoxia and fibrosis. Hyperoxia induces primary neovascularization, secondary growth of healthy granulation tissue, and induces short-term vasoconstriction, which may help control active bleeding [28,29]. It is the most widely reported therapeutic technique in the management of hemorrhagic radiation cystitis. Dellis et al. evaluated the benefit of HBOT in 38 patients with severe radiation cystitis. The complete response rate was 86.8%, and the partial response rate was 13.2%. The mean follow-up was 29.3 months. For the thirty-three patients with complete response who received HBO therapy within 6 months of the hematuria onset, the mean time interval was 4.9 months (range 1–6), while in the remaining five patients with partial response, the mean time interval was 22 months (range 8–48) (*p* < 0.001). Thirty-three patients were alive at the end of follow-up [29].

Recently, the randomized phase 2–3 RICH-ART evaluated the benefit of HBOT compared to standard of care for patients with late radiation cystitis and a value of fewerthan 80 points in the urinary domain of the expanded prostate index composite score (EPIC score). Forty-one patients were randomized in the HBOT arm and 38 in the standard of care arm. HBOT significantly alleviated patient-perceived symptoms of late radiation cystitis and improved HRQOL. The mean improvement in EPIC urinary total score was higher (17.8 [SD 18.4]) in the hyperbaric oxygen therapy group compared with patients in the control group (7.7 [SD 15.5]). Seventy patients in HBOT presented a grade 1–2 adverse events. The main adverse events grade 1–2 were ear pain (15%), myopia (12%) and barotrauma (10%). No grade 3–4 or 5 was reported in this group [30]. The HBOT’s benefit was maintained in the time. In fact, Pereira et al. reviewed 105 patients diagnosed with RIHC whowere treated with HBOT between 2007 and 2016. After a median follow-up of 63 months, 76.3% had a complete response [31]. Cardinal et al. evaluated the benefits of HBOT through a meta-analysis of data from 602 patients treated with HBOT for hemorrhagic radiation cystitis. They determined that 84% of patients achieved partial or complete resolution, while 75% saw an improvement in hematuria. In their analysis of 499 patients with documented follow-up, authors observed a recurrence rate of 14%, with a median time to recurrence of 10 months (6 to 16.5 months). To summarize, this treatment is well-tolerated, the most common side effects being pressure-related, most notably ear and sinus barotrauma. HBOT is offered to patients for whom bladder washings and instillations are ineffective [32]. In a systemic review, Villeirs et al. emphasized HBOT benefit in radiation cystitis. In a cohort of 815 patients, an overall and complete response rate varied from 64.8% to 100% and 20% to 100%, respectively. Blood transfusion before HBOT, other treatment modalities before HBOT, use of anticoagulant therapy, along the interval between the onset of hematuria and start of HBOT were possible factors associated with lower efficacy of HBOT [27]. It is important to start HBOT in the onset of late radiation cystitis symptoms [33,34]. However, the availability and cost-effectiveness of high-pressure oxygen tanks is a critical factor in the success of HBOT [27].

### 3.3. Late Radiation Cystitis with Refractory or Life-Threatening Hematuria

In late radiation cystitis with refractory or life-threatening hematuria, the treatments aim at achieving volume expansion and at limiting the need for frequent transfusions due to active bleeding [35].

#### 3.3.1. Arterial Embolization

Improvements in interventional radiological techniques have led to improvements in morbidity and mortality compared with surgery in patients with refractory hemorrhagic radiation cystitis. The technical success rate reported is 88–100%. The main adverse events were Brown–Sequard’s syndrome, bladder necrosis, and gluteal paresis or skin necrosis. Thanks to improved techniques, the incidence of adverse events has decreased from 65% to 9–31% [36,37]. The follow-up of these studies is brief.

#### 3.3.2. Cystectomy and Urinary Diversion

In some patients, treatment by means of cystectomy with urinary diversion is unfortunately inevitable when clot evacuation, bladder fulguration and bladder irrigation have failed. This therapeutic option should be reserved for patients for whom local and conservative treatments have proven unsuccessful, given its high rate of morbidity and mortality. Linder et al. reported a postoperative complication rate of 42% and a 90-day mortality rate of 16% [38].

## 4. Clinical Trials: Other Therapeutic Avenues, Antifibrotics

### 4.1. Antifibrotic or Antioxidant Pharmacological Agents

Vitamin E has protectiveeffects against oxidative stress and also plays an important role in preventing lipid peroxidation in the cellular membrane [39]. Between April 2003 and July 2009, 53 breast cancer patients were recruited to determine if a combination of Pentoxifylline (PTX) and Vitamin E could prevent the development of radiation fibrosis after radiotherapy for the definitive management (NCT00583700). This clinical study of post-irradiation cancer patients treated with PTX/vitamin E showed a significant difference in radiation-induced fibrosis. Of importance, the combination of PTX/vitamin E did not impact local control or survival within the first 2 years of follow-up (which is still quite a short follow-up). The oral PTX/vitamin E treatment was safe and well-tolerated. After pelvic irradiation in high-risk patients, the combination of Pentoxifylline (PTX) and Vitamin E can thus be considered clinically useful in preventing fibrosis [40,41]. Orgotein copper-zinc superoxide dismutase (SOD) is an enzyme found in various tissues throughout the body and has a fundamental role in the elimination of reactive oxidative species and free radicals that cause tissue damage and fibrosis [42]. SOD was found to be effective in reducing radiation-induced fibrosis by a reduced pain score and a decrease in the size of the fibrotic area in half of the cases after 6 months in 44 patients with clinical radiofibrosis following conservative treatment of breast cancer [43]. However, the role of antifibrotic agents in reducing or mitigating radiation cystitis remains unknown.

### 4.2. Angiotensin-Converting-Enzyme Inhibitors

An initial clinical study had revealed that angiotensin-converting-enzyme inhibitors mightdecrease the incidence of radiation pneumonitis in patients receiving thoracic radiation for lung cancer [44]. An ongoing clinical study (NCT01754909) is evaluating the efficacy of enalapril on radiation pneumonitis and fibrosis for patients undergoing radiation therapy for lung cancer or other intrathoracic cancers. A recently published phase 2 prospective study supported the notion of radio-induced fibrosis reversibility, showing that pravastatin (40 mg/d for 12 months) was an efficient antifibrotic agent in patients with grade ≥ 2 cutaneous and subcutaneous fibrosis following head and neck radiotherapy [45]. However, no data are available in the setting of radiation cystitis.

## 5. Impact of Macrophages in the Development of Radiation Fibrosis

**Figure 2:**
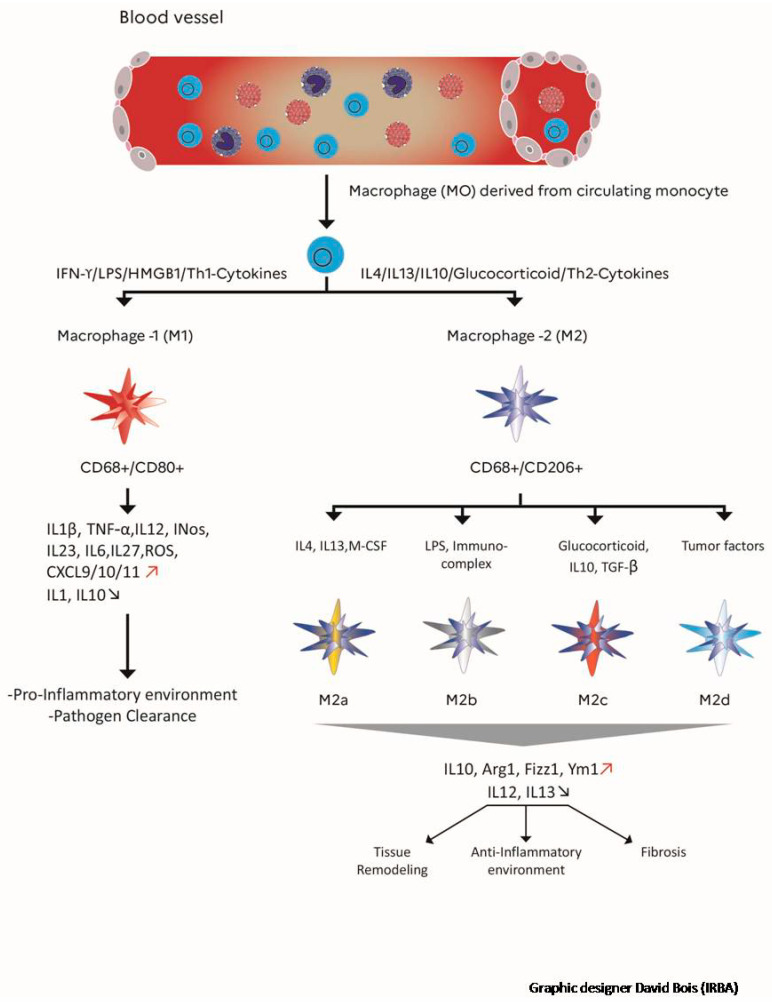
Role of macrophage polarization in inflammation, phenotype markers and signaling molecules involved in M1/M2, M2a, M2b, M2c, M2d. Importance of M1/M2 balance in tissue repair. The M1 CD68 +/CD80 + macrophages which are pro-inflammatory and the M2 CD68 +/CD206 + macrophages which are anti-inflammatory. Red arrows: elevation. Blue arrow: decrease

Recent insights regarding the functional role of inflammatory cells suggest that inflammation could play a role beyond the classical “acute” phase. During the radiation wound repair process, recruitment of inflammatory cells occurs at the site of injury, which can contribute to late inflammatory tissue damage through a continuous mechanismbetween inflammation, hypoxia and fibrosis [46]. During normal healing, sequential activation of the classical, proinflammatory, M1 and alternately activated macrophages, M2a, M2b and M2c, is known to occur, which facilitates the transitions between the inflammatory, proliferative and remodeling phases of the repair process [47]. Thus, macrophage dysfunction or deficient generation can lead to the uncontrolled production of inflammatory mediators and growth factors. This can modify their communications with other cells (epithelial and/or endothelial cells, fibroblasts, progenitors and stem cells) and contribute to a state of persistent injury, which could, in turn, lead to the onset and maintenance of a pathological fibrotic process [48,49]. Macrophages are heterogeneous cells with various phenotypes and functions in part regulated by their micro-environment. Macrophages commonly exist in two (basically defined) distinct subsets, M1 and M2 macrophages, which have different functions and transcriptional profiles (schematically: M1 macrophages are associated with the production of proinflammatory cytokines, while M2 phenotype reprogramming enhances the production of anti-inflammatory cytokines) [50,51,52]. The representation of macrophage subtypes and their different actions are illustrated in Figure 2. The macrophage reprogramming processes, and steps have been reviewed elsewhere. Briefly, those include 1/an amplified macrophage response, which is a direct amplification following exposure to a reprogramming factor (e.g.,INFɣ) but also a cross-amplification consecutive to another factor, such as lipopolysaccharide; 2/a reciprocal suppression of the alternate phenotype (M1/M2); 3/a cascade activation of the reprogramming mechanisms; 4/a feedback phenomenon [53].There are schematically two typical macrophage reprogramming signaling pathways: on the one hand, activation pathways such as JNK, Notch, TLR/NF-κB (p65/p50), PI3K/Akt2, JAK/STAT1, and HIF1αfavor the M1 phenotype; while on the other hand pathways such as PI3K/Akt1, JAK/STAT3/6, TGF-β/SMAD, TLR/NF-κB (p50/p50) and HIF2α are mainly involved in M2 phenotype programming [54]. After a fractionatedirradiation, an abnormal wound healing response occurs, which is characterized by the accumulation of M2 macrophages that promote fibrosis through the production of TGF-β1 [48,55]. Temporal and spatial coordination of myofibroblast activities with inflammatory macrophages is crucial for the controlled healing process and restored homeostasis in injured tissue, such as after irradiation. In this context, the M1 phenotype has also been associated with an antifibrotic effect by releasing MMPs (MMP-9, MMP-12 and MMP-13 that degrade ECM). However, if the injury process persists, fibrosis progresses with the proliferation of myofibroblasts and ECM deposition (such as collagen and fibronectin) in and around inflamed or damaged tissue [56,57]. In this context, M1 macrophages represent the starting point of the profibrotic process. Indeed, M1 macrophages release proinflammatory cytokines and chemokines that indirectly promote the proliferation of myofibroblasts. In this deregulated tissue microenvironment. There is a modulating of the macrophage phenotype, in which M2 macrophages phenotype can be generated by apoptotic bodies accumulated, ECM quality modified and Th2 cytokines stimulation [58]. M2 initially involves anti-inflammatory cells, with the release of IL10, arginase, TGFβ and HO-1 [59]. When the tissue microenvironment homeostasis is deregulated, M2 activation leads to fibrocytes recruitment and proliferation, myofibroblastactivation, and fibroblasts proliferation. In addition to collagen production, pro-fibro-fibrotic genes are transcripted, leading to the secretion of a large number of profibrotic factors such as TGF-B1, PDGF, IL6, IL-13 IL-7 and galactin-3and to an increase in tissue inhibitors of metalloproteinases (TIMPs) expression [55,57,60,61]. Recent data highlighted that the exacerbation of radiation-induced pulmonary fibrosis might depend on the mesenchymal transition of epithelial cells, promoted by the TGF-β-secreting M2 macrophages [62]. Therefore, any change in the M1/M2 balance will have a central role in terms of fibrosis control or worsening. In addition, exposure to irradiation may also activate macrophages indirectly. An abnormal wound healing response occurs, which is characterized by the accumulation of M2 macrophages, which promote fibrosis through the production of TGF-β1 [63]. Non-coding RNA appears to be involved in the initiation and progression of radiation-induced lung fibrosis by modulating the M2-mediated signaling pathway [64]. In animal models of lung fibrosis, it was reported a high expression of let-7i and low expression of miR-21 at 3 weeks post-irradiation. At a later stage (after 26 weeks), let-7i expression decreased, and miR-21 was upregulated. In addition, it has been described that let-7i targets TGFBR1, inhibiting TGF-β signaling, while miR-21 degrades the TGF-β inhibitor SMAD7. In support of this, the miR-21 expression is upregulated in several models of fibrosis [65,66]. Significant functional and temporal differences have been shown among several distinct miRNAs that are found both in the inflammatory phase (immediately post-irradiation) and in the later fibrotic stages [58]. Mukherji et al. proposed that macrophage activation could be a secondary effect of radiation exposure, which may result from cellular damage signals and clearance of radiation-induced apoptotic cells, rather than a direct effect of irradiation [67]. In Oncoimmunology, Meziani and colleagues et al. provide a thorough discussion of the immune system/macrophage responses to radiotherapy and their involvement in the development of radiation injury. Macrophages are described as a promising therapeutic target for the prevention or the treatment of radiation-induced toxicities [68]. This immunomodulatory approach aims not only to increase the antitumor efficacy of radiotherapy but also to limit its side effects. To limit the initiation of fibrosis in healthy irradiated tissue, the migration of type 2 macrophages or the reprogramming of M1/M2 must be controlled [68]. However, the dynamics of myeloid cells in the bladder after pelvic radiotherapy have not yet been elucidated and must be investigated.

## 6. Preclinical Studies of Radiation Cystitis and Cell Therapy: A New Therapeutic Avenue

### 6.1. Preclinical Studies of Radiation-Induced Cystitis

Animal models of radiation cystitis are preferably performed in rodents. As illustrated in Table 1, the radiation exposure is mostly delivered as a single dose via an X-ray or gamma-ray irradiator. However, it appears more appropriate to use X-rays to study the effects of radiotherapy on tissues. For example, the SARRP (Small Animal Radiation Research Platform) is one of the X-ray irradiators used in preclinical research. These small animal radiotherapy devices enable state-of-the-art image-guided therapy (IGRT) research to be performed by combining high-resolution cone-beam computed tomography (CBCT) imaging with an isocentric irradiation system [69]. This radiation fractionation is, however, not clinically relevant, as most pelvic therapeutic irradiations are delivered through fractionated schemes in patients. Among the factors inducing fibrosis, Th-2 cytokines were among the first to be recognized to have strong profibrotic properties. Typical cytokines released from Th-2 cells are IL-4, IL-5, IL-10 and IL-13. Three of them, IL-4, IL-5 and IL-13, are linked to fibrosis development [70]. A few months after irradiation of the bladder, degenerative epithelial tissue, urothelial swelling, pseudo-carcinomatous epithelial hyperplasia, fibrous tissue in the lamina propria and between muscle cells, a mild increase in inflammatory cells, disruptions in tight junction formation, edema, loss of endothelial cells, urothelial hyperplasia, and bleedings (in the most severe cases) were detected [71]. As described in Table 1, three preclinical treatments were shown to reduce the development of radiation cystitis, including Hyperbaric oxygen therapy (HBOT), liposomal and tacrolimus instillations and also vasculogenic and angiogenic localized therapies [72,73,74]. For HBOT, 14 days after radiation, rats were treated in the chamber (95% oxygen and pressurized to 200 kPa for 90 min), twice daily, for a period of two weeks. This therapy reduced radiation oxidative stress and TGF-Beta and consequently lowered levels of IL-10. Using endothelial cells as a vasculogenic therapy and vascular endothelial growth factor (VEGF) as an angiogenic therapy wasbeneficial in the early chronic phase. But this angiogenic therapy using endothelial cells could promote tumor revascularization, although routine endothelial cells culture is still limited [75,76]. It is well documented that ionizing radiations activate the Nuclear Factor κB (NF-κB) signaling cascade directly or via induction of double-strand breaks and oxidative stress [77]. The NF-κB pathway is a link to the immune system in radiation response [78]. Thalidomide, an immunosuppressive drug thatinterferes with the activation of NF-κB, may be a valid treatment option for patients with inflammatory diseases refractory to other first- and second-line treatments. Considering the immunomodulatory effect of thalidomide, Kowaliuk et al. recently investigated the role of NF-κB and the functional effects of this treatment on radiogenic bladder dysfunction. Early thalidomide infusion after pelvic irradiation using a YXLON MG325 X-ray device showed beneficial and promising effects on the incidence and severity of bladder dysfunction [79]. The late administration of thalidomide showed no significant effect on functionality with possible neurological side effects, limiting its use [80]. Oral administration of clarithromycin or isoflavone before and after irradiation results in the anti-inflammatory macrophage subtype switch and reduction of macrophage infiltrate, respectively [81,82]. Intraperitoneal injection of Melatonin before radiation reduces lymphocytic and macrophagic infiltrates [83]. Moreover, Intraperitoneal injection over 8 weeks of Purified murine anti-IL-13 IgG antibody, starting 3 Week post radiation exposure participate ininhibition of polarization of alternatively M2-macrophages, also after Iterative IV infusion 5 × 10 Adipose -MSCs/infusion [84,85]. To increase the chances of finding a potential preclinical treatment for radiation cystitis, it is imperative to explore novel mitigators of radio-induced inflammatory reactions.

### 6.2. Stem Cell Therapy: A New Therapeutic Avenue

Because of their ability to migrate to the irradiated site and of their immunomodulatory and antioxidant properties in promoting tissue repair, mesenchymal stem (or stromal) cells (MSCs) are a potential antifibrotic therapeutic candidate [86,87,88,89,90]. Preclinical studies have described their beneficial effects, in particular their ability to limit the development of pulmonary and colorectal after irradiation by modulating the polarization of macrophages. From these investigations, it seems that MSCs could not only replace damaged epithelial cells but also promote tissue repair through the secretion of anti-inflammatory and antifibrotic factors [85,91,92]. However, it is important to note that these studies were performed on non-cancerous models. In a recent preclinical study of radiotherapy to treat colorectal cancer, it was shown that treatment with bone marrow (BM)-derived MSCs significantly reduced both cancer initiation and cancer progression by increasing the number of tumor-free animals as well as decreasing the number and the size of the tumors by half, thereby extending their lifespan. The attenuation of cancer progression was mediated by the capacity of the MSCs to modulate the immune component. The MSCs reprogrammed the macrophages to become regulatory cells involved in phagocytosis, thereby inhibiting the production of proinflammatory cytokines. Thus in the long term post-radiotherapy, this biotherapy allows the maintenance of tissue homeostasis and inhibits tumor progression [93]. MSCs inhibit fibrosis by reducing the expression of TGF-𝛽1, modulating the inflammatory response, apoptosis, oxidative stress and remodeling of the extracellular matrix. In particular, preclinical studies have shown that MSCs could act on fibrosis by directing the polarization of macrophages and the differentiation of CD4+ T lymphocytes [94,95,96]. In response to signals derived from tissue damage, macrophages undergo reprogramming, which leads to the emergence of a spectrum of distinct functional phenotypes (Figure 2). A study by Chen et al. showed that MSCs couldpromote M2 macrophage polarization by secreting TGF-β3 and TSP1 [97]. Recent publications have shown that MSCs could induce M2 macrophages through the secretion of exosomes, and these effects could be due to the activation of transcription factors Stat6, MafB [98] and the secretion of miR-223 targeting PKNOX1 in macrophages [99]. These regulatory mechanisms are involved in acute inflammation. However, in the case of chronic radiation cystitis, fibrosis is triggered by chronic inflammation. MSCs could inhibit chronic inflammation by altering the polarization of macrophages to resolve chronic inflammation through the secretion of exosomes containing miR let-7b [100]. Moreover, HGF and TSG-6 have been shown to be major effectors of the antifibrotic activity of MSCs in several models (e.g., cutaneous and renal fibrosis [101,102,103]. HGF has been shown to be up the urine of prostate cancer survivors with a radiation history [104]. HGF could potentially play a dual role in radiation cystitis whereby it promotes angiogenesis and is protective against fibrosis [105]. TSG-6 is able to form hyaluronan polymers, which trigger the activation of NF-κB and the subsequent acquisition of the M1 phenotype [82]. Thus, TSG-6 could act as a negative regulator of M2 activity by promoting the availability of hyaluronan. As prolonged M2 activity has previously been associated with worsening fibrosis, newly secreted TSG-6 could be a major regulator of inflammation after MSC transplantation [106,107].

## 7. Discussion and Conclusions

Although irradiation techniques have improved over time, the incidence of radiation cystitis still poses a real problem for clinical management. Indeed, the management of radiation cystitis, especially in the late-stage, is based largely on symptomatic treatments. This was historically explained by the (theoretical) irreversibility of late-stage histological fibrotic lesions. Despite encouraging results, evidence that radiation cystitis can be modulated pharmacologically is insufficient and requires further confirmation as these findings are based only on small sample sizes or on retrospective analyses. The pathogenesis of fibrotic diseases remains a major challenge, due not only to the variety and multiplicity of initiating events but also to a large number of profibrotic mediators involved. Nevertheless, MSCs appear to be a promising therapeutic alternative for the treatment of fibrosis in chronic radiation cystitis. The pro- or antitumor effects of biotherapies using MSCs have been widely discussed in the literature and are one major parameter that must be better understood before clinical application [108,109,110]. Other stem cells may be promising treatments of hemorrhagic cystitis. The placenta is a potential source of stromal cells, with decidual stromal cells (DSCs). These stem cells are easily amplified in vitro and have greater immunosuppressive potential than BM-MSCs. DSCs inhibit alloreactive T cell proliferation better than stromal cells from other sources and induce coagulation more effectively than BM-MSCs. Iterative infusions can be considered in patients with inflammatory pathologies [111,112,113]. In preclinical studies, compared to bone marrow-derived MSCs, DSCs had better viability [114]. Their clinical use must, however, be optimized. It should be noted that stromal cell injections must be carried out only in a patient with a long complete remission to limit their potential implantation near dormant cancer cells. Such safety issues may limit the use of MSC in clinics. MSC paracrine action is widely described in the literature [115,116]. To limit the implantation of these cells, it is possible to use the extracellular microvesicles (Evs-MSCs) that they secrete to reduce radiation-induced lesions, including fibrosis, without exposing patients to the risk of cancer reactivation [117,118,119].

To increase the likelihood of finding a potential preclinical treatment for radiation cystitis, it is imperative to test novel mitigators of the radio-induced inflammatory reaction. For example, TCDO/WF10 is a chemically stabilized chlorite matrix that has previously been shown to have a positive effect in the context of chronic inflammatory conditions. It induces natural immunity and stimulates cellular defense mechanisms through its actions on natural killer cells, cytotoxic T lymphocytes, and modification of the monocyte-macrophage system. It reduces inflammation quickly so that healing can begin [120,121]. In an early-stage clinical study, 20 patients with grade 3 radiation cystitis received intravenous TCDO treatment for 5 consecutive days. From 1 to 9 months after TCDO treatment, patients had no recurrent bleeding, and no side effects from treatment with TCDO were observed [122]. In a phase-II study involving 100 patients, Veerasarn et al. evaluated the effectiveness of WF10 in combination with standard care compared to standard care alone. The complete resolution rate for hematuria was comparable in both arms (74% vs. 64% in the experimental arm and in the standard arm, respectively). However, a significant reduction in the relapse of hematuria was noted among responders in the experimental arm (47% vs. 77%, *p*-0.01). No severe adverse events were reported [123,124].

To date, no preclinical treatment without reconstructive surgery appears to fully restore the function and structure of the bladder after radiation exposure. A number of preclinical studies have described strategies for limiting fibrosis (cf. Table 1). The majority of studies have been carried out in mice on a model of radio-induced pulmonary fibrosis. Those were mitigating and continuous treatments (isoflavone mixture gavage, oral clarithromycin) that were evaluated with respect to the inflammation and migration of immune cells, including macrophages, and the development of fibrosis [81,125]. Clarithromycin (CLA) administration, before and after lung radiation exposure, reduced expression levels of TNF-α, TNFR1, TNFR2, TGF-β1, CTGF and type I collagen, and inhibits both the increased acetylation of NF-κB p65 and the elevated expression of COX-2 with reduction of both fibrosis and macrophage infiltration [81]. To reduce fibrosis, as described by Chung et al., it is necessary to target type 2 cytokines such as IL13, limiting recruitment and polarization of Ym1/Chi3L3-positive macrophages, which are alternatively activated in the lungs following thoracic irradiation. Intraperitoneal injection of IL-13 neutralizing antibody does not completely suppress radiation-induced TGF-β expression, suggesting that sustained IL-13 or TGF-β neutralization therapy may be necessary to durably mitigate fibrotic progression [84]. Rapamycin is a potent immunosuppressive drug used in solid organ transplantation for the prevention of allograft rejection. In oncology, mTOR (mammalian target of rapamycin) inhibitors are currently being evaluated in several types of cancers. Targeting mTOR signaling may provide a therapeutic option for radiation-induced lung injury. Indeed, mTORC2 activity has been described in the non-canonical signaling of TGF-β, and mTORC2 inhibitors could thus be of interest in the case of fibrosis [126]. In rats, melatonin or metformin administration before irradiation using a ^60^Co source of gamma rays in the thoracic area (i.e., heart and lungs) helped prevent the infiltration of macrophages and lymphocytes, as well as the upregulation of IL-4, IL4ra1, Duox1, andDuox2 [83,127]. Furthermore, metformin treatment could stimulate the activity of antioxidant enzymes such as superoxide dismutase (SOD) and glutathione (GSH) [128]. Other antioxidants, such as fucoidan, have been orally administered preclinically after whole lung irradiation at 10Gy, mitigating different proteins (TIMP-1, CXCL1, MCP-1, MIP-2, and IL-1Ra) expression in pleural fluid, decreasing pleural fluid accumulation and reducing neutrophil and macrophage infiltration in lung tissues. Fucoidan changed the expression patterns of inflammatory cytokines, which may consequently attenuate lung fibrosis [129,130].

Many molecular mechanisms still need to be better understood in order to develop a targeted treatment for fibrosis and radiation cystitis. It is, therefore, necessary to broaden our knowledge of myeloid and lymphocytic dynamics in the development of this fibrosis induced after pelvic radiotherapy, based on data obtained in other models (ex: lung fibrosis). It is also necessary to follow a step-by-step translational development to ensure that patient outcome may be improved, through the integration of robust biomarkers of toxicity, as well as through the implementation of modern radiotherapy tools in clinical research to minimize the doses to organs at risk, including the bladder, and therefore improve the therapeutic index. The current knowledge on the biological processes involved in late radiation toxicity clearly suggests that it is indeed easier to prevent late toxicity than to reverse [131].

## Figures and Tables

**Table 1:** Recent animal models for preclinical studies of radiation cystitis (RC) and preclinical studies targeting immune cells to limit the development of radio-induced fibrosis.

Animals	Method of Radiation Cystitis Induction	Treatments		Effect(s)	References
		Administration Type	Time Post-Radiation Exposure (PE)		
Adult female Sprague–Dawley rats	Single 20 Gy dose by a linear accelerator (6MV)	20 sessions of HBOT over a fortnight	14 days PE	Reduction of oxidative stress and proinflammatory factors	Oscarsson N et al., 2017
Adult female Sprague–Dawley rats	SARRP, singledose 40 Gy 6–8 weeks PE: histological tissue damage to the bladder	Liposomal tacrolimus instillation	6 weeks PE	Increase in inter-micturition intervals	Rajaganapathy BR et al., 2015
Adult female Lewis rats	A single 20 Gy dose of using a cesium isotope-based irradiator.	Injection into the bladder wall of a solution containing VEGF +/− endothelial cells	30 days PE	Revascularization of radiation-damaged urinary bladders	Soler R et al., 2011
Female BALB/c mice	Single 10 Gy dose by Siemens Stabilipan X-ray to the whole lung, Thickened alveolar septa, reflective of pneumonitis at 18 weeks PE	Isoflavone mixture gavage	Before and after radiation exposure	M1 subtype switched to an anti-inflammatory M2 subtype with increased levels of Arg-1 and decreased NOS2	Abernathy LM et al., 2015
Female C57BL/6J mice	Single 18 Gy dose by linear accelerator (21EX 3153 VARIAN) to the whole lung Interstitial edema and fibrosis sections at 16 weeks PE,	Oral clarithromycin	Before and after radiation exposure, and continuing until the day of sacrifice	Inhibition of fibrosis scoring, influx of macrophages and interstitial edema	Lee SJ et al., 2015
C57BL/6 female mice	5 × 6 Gy thoracic irradiation by X-RAD 320, Macrophage accumulation in the irradiated lung at 10 weeks PE	Purified murine anti-IL-13 IgG antibody by intraperitoneal (ip) injection	Weekly ip injection over 8 weeks, starting 3 weeksPE	Inhibition of recruitment and polarization of alternatively activated YM-1 positive macrophages	Chung SI et al., 2016
Adult male Wistar rats	^60^ Co source Single dose Gy 15 to the whole lung - Mild fibrosis at 17 weeks PE	- 1 mL of melatonin solution (100 mg/kg) - Intraperitoneal injection	30 min before irradiation	Increased levels of IL-4, DuoX1, Duox-2 and decreased lymphocyte and macrophage infiltration	Aliasgharzadeh A et al., 2019
Sprague-Dawley rats	Single 27 Gy dose by ^60^ Co irradiator inthe colorectal region, Anastomosis in the colon at 4 weeks PE	Iterative IV infusion 5 × 10 adipose-MSCs/infusion	3 weeks PE	The proportion of anti-inflammatory M2 macrophages grew, favoring the M2 phenotype and promoting wound healing	Van de Putte D et al., 2017
